# Advantages of FBPA PET in evaluating early response of anti-PD-1 immunotherapy in B16F10 melanoma-bearing mice: Comparison to FDG PET

**DOI:** 10.3389/fonc.2022.1026608

**Published:** 2022-12-22

**Authors:** Mitsuaki Tatsumi, Fumihiko Soeda, Sadahiro Naka, Kenta Kurimoto, Kazuhiro Ooe, Hideyuki Fukui, Daisuke Katayama, Tadashi Watabe, Hiroki Kato, Noriyuki Tomiyama

**Affiliations:** ^1^ Department of Radiology, Osaka University Hospital, Suita, Osaka, Japan; ^2^ Department of Nuclear Medicine and Tracer Kinetics, Osaka University Graduate School of Medicine, Suita, Osaka, Japan; ^3^ Department of Radiology, Osaka University Graduate School of Medicine, Suita, Osaka, Japan

**Keywords:** immune checkpoint inhibitor, melanoma, mouse, FBPA, FDG, PET

## Abstract

**Purpose:**

PET with L-4-borono-2-[^18^F] fluoro-phenylalanine (FBPA) was reported to be useful to differentiate malignant tumors and inflammation. Although immunotherapy with immune checkpoint inhibitors (ICIs) has been applied to cancer treatment recently, FDG PET may not be suitable to determine the effect of ICIs because of false-positive findings caused by treatment-related inflammation. In this study, we aimed to demonstrate that FBPA PET allowed detection of the early response of anti-PD-1 immunotherapy in tumor-bearing mice, comparing the results with those of FDG PET.

**Materials and methods:**

Mice with B16F10 melanoma tumor xenografts were prepared. Anti-mouse PD-1 antibody or PBS was administered twice intraperitoneally to the tumor-bearing mice on Day 0 (3 days after inoculation) and Day 5 (treatment or control group <TrG or CoG>). PET/CT imaging was performed twice for each mouse on Day 0 before the anti-PD-1 antibody/PBS administration and on Day 7 using a micro-PET/CT scanner. FBPA and FDG PET/CT studies were conducted separately. SUVmax and the tumor to liver ratio (T/L ratio) were used as parameters exhibiting tumor activity. Tumor uptake volume (TUV) and metabolic tumor volume (MTV) were also calculated for FBPA and FDG, respectively. Changes between pre- and posttreatment SUVmax or T/L ratio were observed using the formula as follows: [(posttreatment parameter values/pretreatment values - 1) × 100] (%).

**Results:**

Tumors in TrG were smaller than those in CoG on Day 7. SUVmax and T/L ratio represented no differences between TrG and CoG in FBPA and FDG PET before treatment. FBPA PET on Day 7 demonstrated that SUVmax, T/L ratio, and TUV in TrG were statistically smaller than those in CoG. %T/L ratio and %SUVmax exhibited the same trend in FBPA PET. However, FDG PET on Day 7 revealed no differences in all parameters between TrG and CoG. T/L ratio and %SUVmax in TrG represented larger values than those in CoG without statistical significances.

**Conclusion:**

This study demonstrated that FBPA PET allowed detection of the early response of anti-PD-1 immunotherapy in B16F10 melanoma-bearing mice. FDG PET did not detect the response. Further studies are required to determine whether FBPA PET is useful in evaluating the treatment effect of ICIs in humans.

## Introduction

Cancer immunotherapy with immune checkpoint inhibitors (ICIs) has increasingly been recognized as a novel effective treatment recently.

However, the treatment response is unsatisfactory for most patients as the response rate is limited to about 20% - 40% (1, 2). Methods to predict or determine the efficacy at an early stage of treatment are highly desired due to its high cost and possible autoimmune-like side effects named as immune-related adverse events (irAEs).

Positron emission tomography (PET), a functional and metabolic imaging technique, is a promising candidate of the assessment method in view of evaluating the disease activity of primary and metastatic malignant lesions. PET using [^18^F] fluorodeoxyglucose (FDG PET) has widely been used for tumor imaging since glucose metabolism is enhanced in various types of malignancies. However, FDG PET may not be suitable to determine the effect of cancer immunotherapy at an early stage of treatment because of the false-positive findings caused by inflammation. Theoretically, cancer immunotherapy is accompanied by inflammation in the treatment area and glucose metabolism is enhanced by the inflammation in addition to tumor activity, which is visualized as increased FDG uptake. irAEs caused by ICIs include inflammation in various organs as well. Mekki, et al. reported that thoracic sarcoid-like reaction, enterocolitis, thyroiditis, hypophysitis, and pancreatitis were observed as irAEs in FDG PET (3). These inflammatory changes potentially provide false-positive FDG PET findings.

PET with L-4-borono-2-[^18^F]fluoro-phenylalanine (FBPA), an amino acid-based radiotracer, has been used for pretreatment assessment before boron neutron capture therapy (BNCT) for cancer (4). BNCT is a type of radiotherapy based on the nuclear reaction of [^10^B] (n, α) [^7^Li]; a neutron beam from a nuclear reactor or accelerator is irradiated around the ^10^B containing tumor target and the emitted alpha particles have a higher cytotoxic effect and shorter range than beta rays. L-paraboronophenylalanine (BPA) labeled with ^10^B is the major carrier compound used to deliver the boron selectively to the tumor cells (5). A recent study demonstrated that BPA was delivered to the cells through transporter-mediated mechanisms and that L-type amino acid transporter 1 (LAT1) was the major amino acid transporter related to these mechanisms (6). Another recent study reported that FBPA accumulated into tumor cells mainly *via* LAT1 and that FBPA uptake was significantly lower than FDG uptake in inflammatory lesions (7). FBPA is considered as a promising tumor-specific PET tracer.

In the present study, we aimed to demonstrate that FBPA PET was useful for evaluating the early response of anti-PD-1 cancer immunotherapy in B16F10 melanoma-bearing mice, comparing the results with those of FDG PET. An increase in FDG uptake was shown with anti-PD-1 treatment in a previous study using the same experimental model as this study (8).

## Materials and methods

### Animal model

C57BL/6JJmsSlc mice (6 - 7 weeks old) were purchased from Japan SLC, Inc. (Hamamatsu, Japan). B16F10 melanoma cell line was obtained from ATCC (Manassas, VA, USA). Cells were cultured in Dulbecco’s modified Eagle medium supplemented with 10% fetal bovine serum (FBS), 1% penicillin-streptomycin, and 2 mmol/L L-glutamine.

The mice were inoculated subcutaneously with 3 ×10^6^ B16F10 cells in 100 μL of phosphate-buffered saline (PBS) 3 days before pretreatment PET imaging on Day 0.

### Anti-PD-1 treatment

Anti-mouse PD-1 antibody (clone RMP1-14) was purchased from BioXcell (West Lebanon, NH, USA). The antibody (250 μg) in 200 μL PBS was administered twice intraperitoneally to the tumor-bearing mice on Day 0 and Day 5. Mice with anti-PD-1 antibody were defined as the treatment group. In addition to the treatment group, the control group was prepared, which consisted of mice receiving 200 μL PBS without anti-PD-1 antibody twice intraperitoneally on Day 0 and Day 5.

Tumors were measured using calipers, and the tumor volume expressed in mm^3^ was calculated according to the following formula: 0.5 × (long diameter) × (short diameter)^2^. Relative tumor volume, the volume ratio of posttreatment to pretreatment tumor, was also calculated in this study.

### Synthesis of [^18^F]FBPA

[^18^F]FBPA was synthesized according to previous report (9). In short, [^18^F]FBPA was produced by reacting a precursor solution (30 mg of 4-borono-L-phenylalanine in 4 mL trifluoroacetic acid) with [^18^F]acetylhypofluorite. After the reaction, [^18^F]FBPA was separated by using High-Performance Liquid Chromatography (HPLC), and the HPLC solvent was removed before [^18^F]FBPA was recovered with saline. The radiochemical purity of [^18^F]FBPA was > 98% and the molar activity was 232 GBq/mmol on average.

### PET imaging

PET/computed tomography (PET/CT) imaging was performed twice for each mouse on Day 0 before the anti-PD-1 antibody administration and on Day 7 using a micro-PET/CT scanner (Inveon, Siemens, Munich, Germany).

FBPA or FDG PET/CT studies were conducted separately in 12 tumor-bearing mice (n = 6 each for the treatment and control groups).

FBPA or FDG was injected *via* the tail vein of each mouse (FBPA: 2.18 ± 0.34 MBq, FDG: 2.17 ± 0.25 MBq). Static PET images were acquired for 10 min starting at 60 min after radiotracer injection under isoflurane anesthesia.

PET images were reconstructed using three-dimensional ordered-subset expectation–maximization algorithm (16 subsets, 2 iterations) with attenuation and scatter correction.

### Image analysis

The radioactivity of each tumor was expressed quantitatively as the standardized uptake value (SUV), which was corrected for the injected dose (MBq) and body weight (g). The maximum value of SUV (SUVmax) was calculated from a single voxel exhibiting the maximum SUV in each tumor. The mean of SUV (SUVmean) was obtained in the liver as a background value.

Spherical volumes of interest (VOIs) were placed on the tumor and liver in the PET images using commercially available software (PETSTAT, AdIn Research, Tokyo, Japan) while referring to the CT images.

The tumor to liver ratio (T/L ratio) was also used as a parameter exhibiting tumor activity, which was defined by the ratio of SUVmax in the tumor to SUVmean in the liver. In addition to pre- or posttreatment parameters in the tumor, changes between pre- and posttreatment tumor activity were observed using the formula as follows: [(posttreatment parameter values/pretreatment values - 1) × 100] (%). They were expressed as %SUVmax and %T/L ratio.

Tumor uptake volume (TUV) and metabolic tumor volume (MTV) were also calculated for FBPA and FDG, respectively. TUV or MTV was defined as the volume within a tumor margin, which was delineated with 40% of SUVmax. These quantitative parameters were also obtained from the same software mentioned above.

### Immunohistochemistry

After the mice were sacrificed by euthanasia, tumor xenografts were resected and subjected to immunohistochemical staining. Immunohistochemical staining was performed to determine if LAT-1 and GLUT-1 transporters, respectively, for FBPA and FDG uptake changed between the treatment and control groups. CD8 and PD-1 proteins were also evaluated in the two groups to observe their changes after treatment.

The antibodies used in this study were anti-LAT1 antibody (orb96302, Biorbyt, Cambridge, UK) for FBPA, anti-GLUT-1 antibody (ab115730, abcam, Cambridge, UK) for FDG, anti-PD-1 antibody (#84651, CST, Danvers, MA USA) for PD-1 expression, and anti-CD8 antibody (14-0808-80, Invitrogen, Waltham, MA, USA) for CD8 expression in accordance with the manufacturer’s instructions. PD-1 and CD8 expressions were evaluated to recognize the association between these markers and FBPA or FDG uptake.

Serial 4-μm tumor paraffin-embedded sections were used for the immunohistochemical staining as well as hematoxylin-eosin (HE) staining.

### Statistical analysis

Unpaired one-tailed t-tests were used to compare the values between the two groups. Statistical significance was set at p < 0.05.

## Results

### Tumor volume

**Figure 1:**
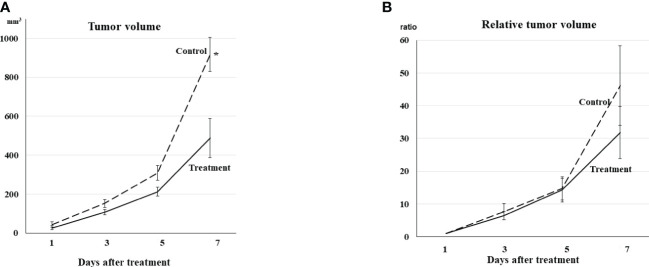
**(A)** Tumor volume Tumor volume increased gradually even in the treatment group. Tumor volumes in both groups showed no differences on Days 3 and 5. The tumors in the treatment group were statistically smaller than those in the control group on Day 7 (*p <0.01). **(B)** Relative tumor volume Relative tumor volume to the volume on Day 1 exhibited the same trend from Day 1 to Day 7 as actual tumor volume, although no statistical difference was observed between the treatment and control groups on Day 7.

Tumors on Day 1 represented no statistical difference in volume between the treatment and control groups. Tumor volumes in both groups also showed no statistical differences on Days 3 and 5. However, tumors in the treatment group were statistically smaller than those in the control group on Day 7 (p <0.01, **Figure 1A**).

Relative tumor volume exhibited the same trend from Day 1 to Day 7, although no statistical difference was observed between the treatment and control groups on Day 7 (**Figure 1B**).

### PET visual analysis

**Figure 2:**
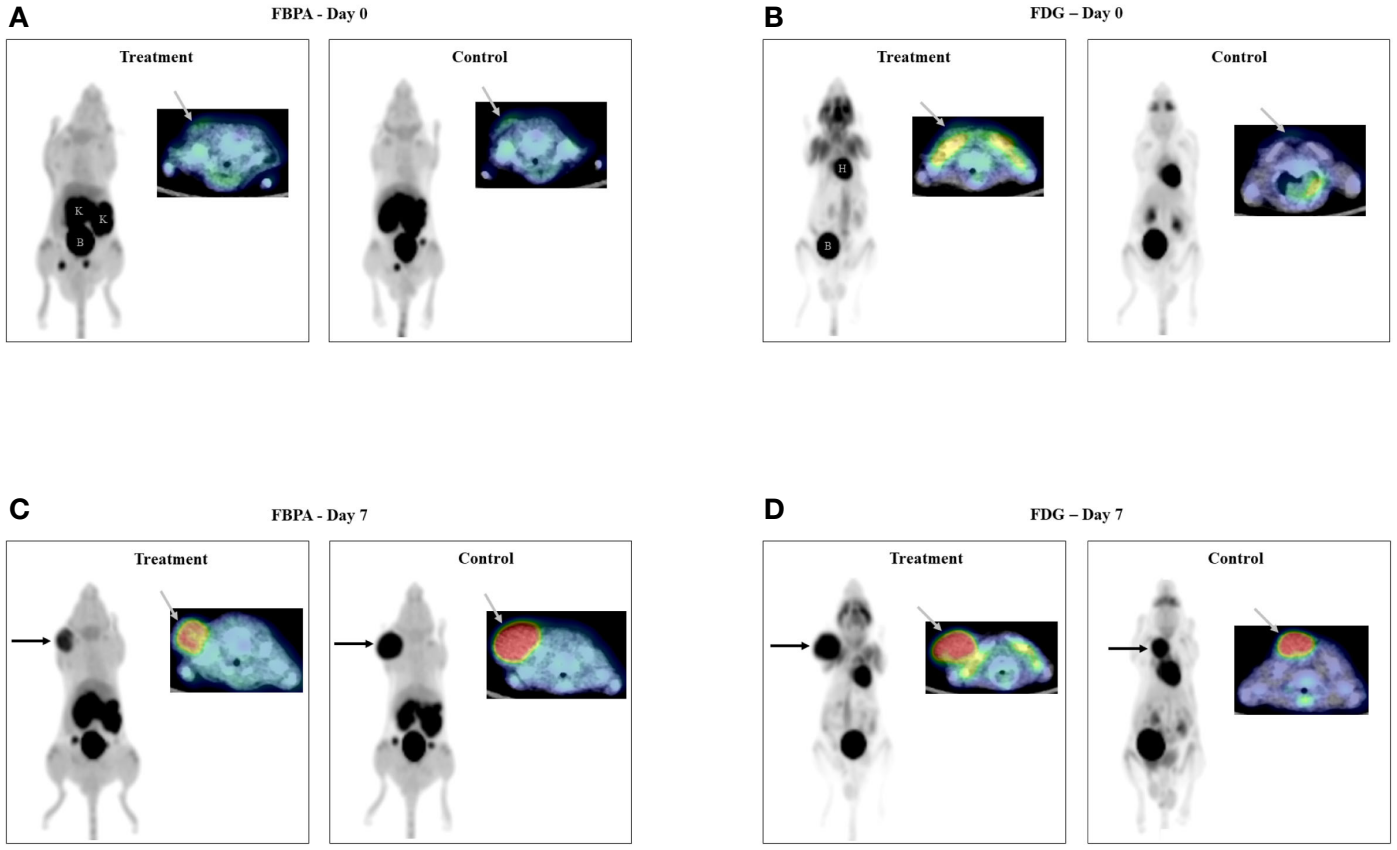
PET/CT images on Day 0 (2**A**: FBPA, 2**B**: FDG) Faint uptake (gray arrow) was observed in all tumors only on transaxial FBPA and FDG PET/CT images before treatment. PET/CT images on Day 7 (2**C**: FBPA, 2**D**: FDG) Intense uptake (black and gray arrows) was observed in all tumors on FBPA and FDG PET/CT images. Visual analysis did not discriminate the tumors in the treatment and control groups either on FBPA or FDG PET/CT images. Radioactivities were observed in the kidneys (K) and bladder (B) and in the heart (H) and bladder (B) in addition to the tumor, respectively, as shown in FBPA and FDG PET images on Day 0. (Left: maximum intensity projection image of PET, right upper: fused PET/CT image, Treatment: PET image in the treatment group, Control: PET image in the control group).

Both FBPA and FDG PET exhibited faint uptake in the tumor before treatment on Day 0 (**Figures 2A**: FBPA, **2B**: FDG).

FBPA and FDG PET showed intense tumor uptake either in the treatment or control group on Day 7 (**Figures 2C**: FBPA, **2D**: FDG). FBPA or FDG uptake was higher on Day 7 than Day 0 in each tumor.

Visual analysis solely did not allow differentiation of the tumors between the treatment and control groups.

### PET quantitative analysis

FBPA and FDG PET before treatment

Both FBPA and FDG PET demonstrated similar SUVmax and T/L ratios of the tumors in the treatment and control groups before treatment on Day 0 without statistical differences. Mean SUVmax and T/L ratio were 0.94 and 0.85 in the treatment group and 0.99 and 0.85 in the control group of FBPA PET, respectively. Mean SUVmax and T/L ratio were 1.0 and 1.7 in the treatment group and 1.1 and 1.4 in the control group of FDG PET, respectively.

### FBPA PET after treatment

**Figure 3:**
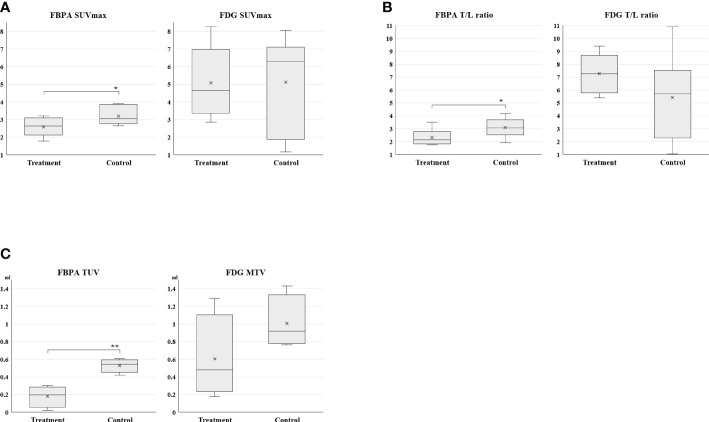
(3**A**: SUVmax, 3**B**: T/L ratio, 3**C**: TUV and MTV) Box charts of quantitative PET parameters in the treatment and control groups Statistically smaller values of SUVmax, T/L ratio, and TUV were observed in the treatment group than the control group on FBPA PET (*p <0.05, **p < 0.005). A substantial overlap was observed in these parameters on FDG PET.

Posttreatment PET on Day 7 demonstrated that SUVmax, T/L ratio, and TUV in the treatment group were statistically smaller than those in the control group (**Table 1**, **Figures 3A**: SUVmax, **3B**: T/L ratio **3C**: TUV). Mean SUVmax, T/L ratio and TUV were 2.6, 2.3, and 0.18 in the treatment group and 3.2, 3.1, and 0.53 in the control group, respectively. %SUVmax and %T/L ratio in the treatment group tended to be smaller than those in the control group without statistical significances (**Table 1**). Mean %SUVmax and %T/L ratio were 183 and 178 in the treatment group and 225 and 267 in the control group, respectively.

**Table 1:** Comparison of quantitative parameters between the treatment and control groups in PET using FBPA or FDG.

	FBPA			FDG		
	Treatment	Control		Treatment	Control	
**SUVmax**	2.6 ± 0.22	3.2 ± 0.2	p <0.05	5.1 ± 0.82	5.1 ± 1.1	n.s.
**T/L ratio**	2.3 ± 0.26	3.1 ± 0.28	p <0.05	7.3 ± 0.62	5.4 ± 1.4	n.s.
**%SUVmax**	183 ± 32	225 ± 24	n.s.	403 ± 43	331 ± 65	n.s.
**%T/L ratio**	178 ± 32	267 ± 38	n.s.	341 ± 33	305 ± 73	n.s.
**TUV or MTV**	0.18 ± 0.061	0.53 ± 0.035	p <0.005	0.61 ± 0.24	1.0 ± 0.15	n.s.

TUV, Tumor uptake volume. n.s., not significant.n.s., not significant.

### FDG PET after treatment

Tumors in both groups exhibited no statistical differences in all parameters again after treatment on Day 7 (**Table 1** and **Figure 3A**: SUVmax, **3B**: T/L ratio, **3C**: MTV). However, T/L ratio and %SUVmax in the treatment group represented larger values than those in the control group in this setting (**Table 1**). Mean SUVmax, T/L ratio and MTV were 5.1, 7.3, and 0.61 in the treatment group and 5.1, 5.4, and 1.0 in the control group, respectively. Mean %SUVmax and %T/L ratio were 403 and 341 in the treatment group and 331 and 305 in the control group, respectively.

### Immunohistochemical analysis

Intense LAT1 and GLUT-1 expression, respectively, for FBPA and FDG uptake was observed in many areas within the tumor, whereas PD-1 and CD8 expression was in the limited areas within the tumor in both the treatment and control groups.

**Figure 4:**
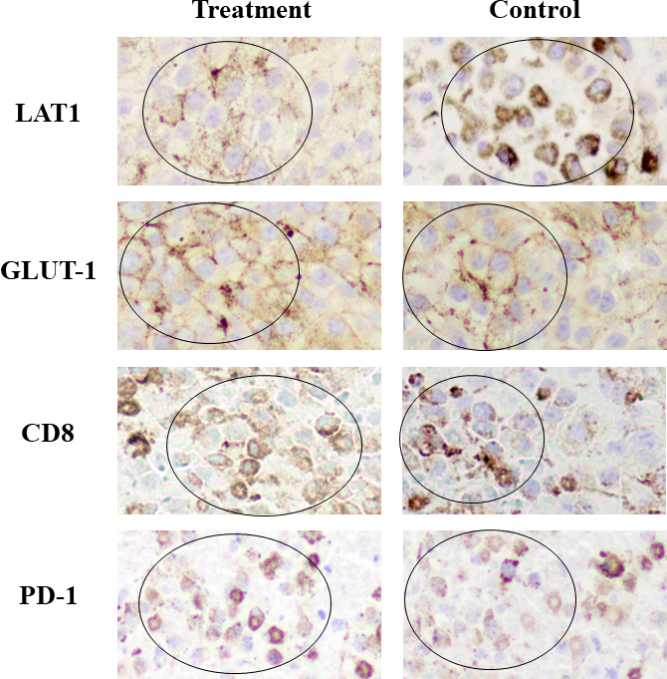
Immunohistochemical staining Intense LAT1 and GLUT-1 expression was observed in many areas within the tumor, whereas CD8 and PD-1 expression was in the limited areas within the tumor in both the treatment and control groups. (Positive staining: mainly observed in circled areas) No differences in the expression of LAT1, GLUT-1, CD8, or PD-1 were observed visually between the treatment and control groups. GLUT-1 and CD8 expression in the treatment group appeared slightly higher than that in the control group.

Visual analysis did not discriminate the expression of LAT1, GLUT-1, PD-1, or CD8 between the treatment and control groups (**Figure 4**).

These results were in line with the PET findings that intense FBPA and FDG tumor uptake was observed either in the treatment or control group on Day 7. Treatment-induced inflammation was not prominent in the model used in this study.

## Discussion

FBPA and FDG were used as radiotracers of PET imaging in this experimental study and discordant findings were observed between them at an early stage of the anti-PD 1 immunotherapy. Although conducted in the tumor-bearing mice, this study firstly compared an amino acid-based radiotracer and FDG in such an immunotherapy setting. The promising findings of FBPA PET in this study warrant further studies of this imaging technique in evaluating the response of immunotherapy with ICIs in humans.

Tumors in the treatment group were smaller than those in the control group on Day 7 in this study. However, all tumors in the treatment and control groups were on the way of growing, and size reduction of the tumors was not observed even in the treatment group. A similar trend of tumor growth was reported in the previous studies dealing with anti-PD-1 treatment in the same tumor-bearing mouse model (8, 10). The slightly smaller tumor volume in the treatment group compared to the control group was considered a reflection of the early treatment effect with the anti-PD-1 antibody in mice with B16F10 melanoma.

FBPA allowed detection of the anti-PD-1 treatment effect on Day 7. SUVmax, T/L ratio, and TUV in the treatment group represented smaller values than those in the control group with statistical significances. %SUVmax and %T/L ratio exhibited a similar trend although statistical significances were not observed. FBPA has been reported to be a tumor-specific PET tracer and shows low uptake in inflammatory lesions (7). Additionally, this study demonstrated that FBPA was useful in evaluating the early response by the anti-PD-1 therapy, which is known to cause immune and inflammatory reactions in the tumor.

Only a limited number of studies have been reported so far regarding the use of amino acid-based PET radiotracers in evaluating the treatment response to cancer immunotherapy with ICIs. Galldiks, et al. reported the additional value of ^18^F FET <(O-(2-[^18^F]fluoroethyl)-L-tyrosine> to contrast-enhanced MRI for treatment monitoring of immunotherapy with ICIs or targeted therapy (TT) alone or in combination with radiotherapy in patients with metastatic brain tumors (11). FET PET seemed to be of great value for the differentiation of treatment-related changes from metastatic brain tumors.

Tomita, et al. reported that anti-PD-1 treatment increased mean FDG uptake values in the tumor in the same tumor-bearing mouse model as this study (8). Maximum FDG uptake values in the tumor represented no statistical difference in their study. In this study, quantitative parameters such as SUVmax, T/L ratio, and MTV in the tumors exhibited no statistical differences between the treatment and control groups. However, T/L ratio and %SUVmax in the treatment group tended to represent larger values than those in the control group. The slight increase in these quantitative parameters appeared to correspond to the increased tumor FDG uptake observed in the study of Tomita, et al. as a reflection of the immune response caused by the anti-PD-1 treatment.

We used SUVmax and T/L ratio to express radiotracer uptake in the tumor in PET quantitative analysis. Although SUVmax or SUVmean has frequently been used as a quantitative parameter in research using PET, it requires the injected dose of radiotracer for calculation. The estimation of actual injected doses is difficult in small animals as the residual radioactivity in tail veins or syringes after injection is relatively large compared to the radioactivity of doses prepared. In this regard, T/L ratio might be more reliable than SUVmax since it is a completely image-derived parameter.

Volumetric FDG PET parameters, MTV and TLG, are known to be better than SUVmax in evaluating or predicting chemotherapeutic responses in clinical situations. Recent studies also demonstrated that volumetric parameters were useful in evaluating the early response by the immunotherapy with ICIs (12, 13). The volumetric parameter, TUV, was also used for FBPA PET in this study. TUV in addition to SUVmax and T/L ratio in the treatment group represented smaller values than those in the control group. However, MTV in FDG PET exhibited no statistical differences between the treatment and control groups. This study successfully demonstrated that volumetric PET parameters were useful in evaluating the early response of anti-PD-1 treatment in the tumor-bearing mice.

IHC analysis demonstrated that intense LAT1 or GLUT-1 expression was observed in many areas within the tumor, whereas CD8 expression was in the limited areas. No obvious differences in these expressions were observed between the treatment and control groups. The clear mechanism of the slight increase in tumor FDG uptake after treatment was not resolved in this study. An experimental study using the same tumor-bearing mouse model as our study demonstrated that anti-PD-1 therapy increased glucose metabolism by cancer cells themselves at an early stage of treatment (8). The subtle inflammatory change caused by anti-PD-1 therapy was not considered to largely affect glucose metabolism in that study.

This study has some limitations. As we would like to demonstrate the advantages of FBPA over FDG in detecting early response of anti-PD-1 immunotherapy, we compared these two radiotracers in the same experimental model as used in the paper dealing with FDG uptake (8). Thus, we used only one treatment protocol with early drug administration, low-dose and short-term regimen. We only used a B16F10 melanoma tumor-bearing mouse model according to the previous studies dealing with anti-PD-1 treatment (8, 10). Thus, the results in this study may not apply to other kinds of tumors in mice or in humans. The observation of tumor growth was limited up to a few days from PET imaging due to an ethical consideration to small animals. An obvious reduction in tumor size was not confirmed in both the treatment and control groups. However, smaller tumor volume in the treatment group compared to the control group was observed and was considered a reflection of the early treatment effect with the anti-PD-1 treatment as stated above. Although discrepant quantitative findings were observed after treatment between FBPA and FDG PET, these findings were obtained from tumors in different mice. Direct comparison of the FBPA and FDG findings in the same tumor would have been ideal, but it was impossible as both FBPA and FDG are ^18^F labeled radiopharmaceuticals.

## Conclusion

This study demonstrated that FBPA PET allowed detection of the early response of anti PD-1 immunotherapy in B16F10 melanoma-bearing mice. FDG PET did not detect the response. Further studies are required to determine whether FBPA PET is useful in evaluating treatment effect of ICIs in humans.

## Data availability statement

The raw data supporting the conclusions of this article will be made available by the authors, without undue reservation.

## Ethics statement

The animal study was reviewed and approved by Animal Care and Use Committee of the Osaka University Graduate School of Medicine.

## Author contributions

The study was designed by MT and FS. All authors participated in collecting data. MT and HF prepared the manuscript and contributed to data analysis and interpretation. All authors contributed to the article and approved the submitted version.
