# Supplementary figures and images for: Advantages of FBPA PET in evaluating early response of anti-PD-1 immunotherapy in B16F10 melanoma-bearing mice: Comparison to FDG PET
Source: Front Oncol. 2022 Dec 22;12:1026608. doi: 10.3389/fonc.2022.1026608 (PMC9815495; doi:10.3389/fonc.2022.1026608)

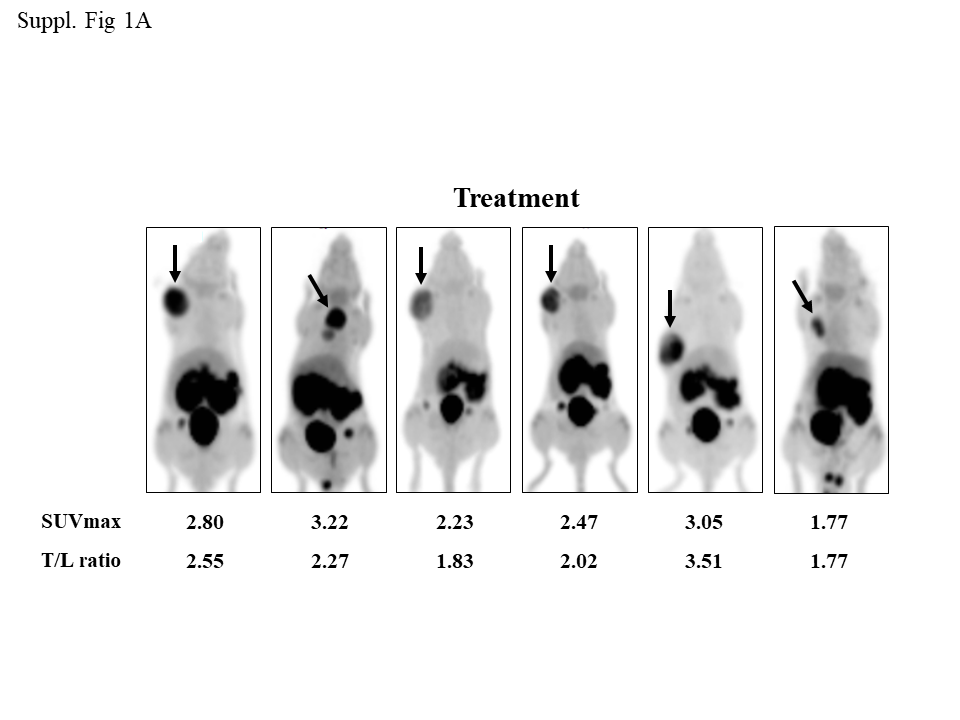

Supplement: Supplementary file 1 [file Image_1.tif]

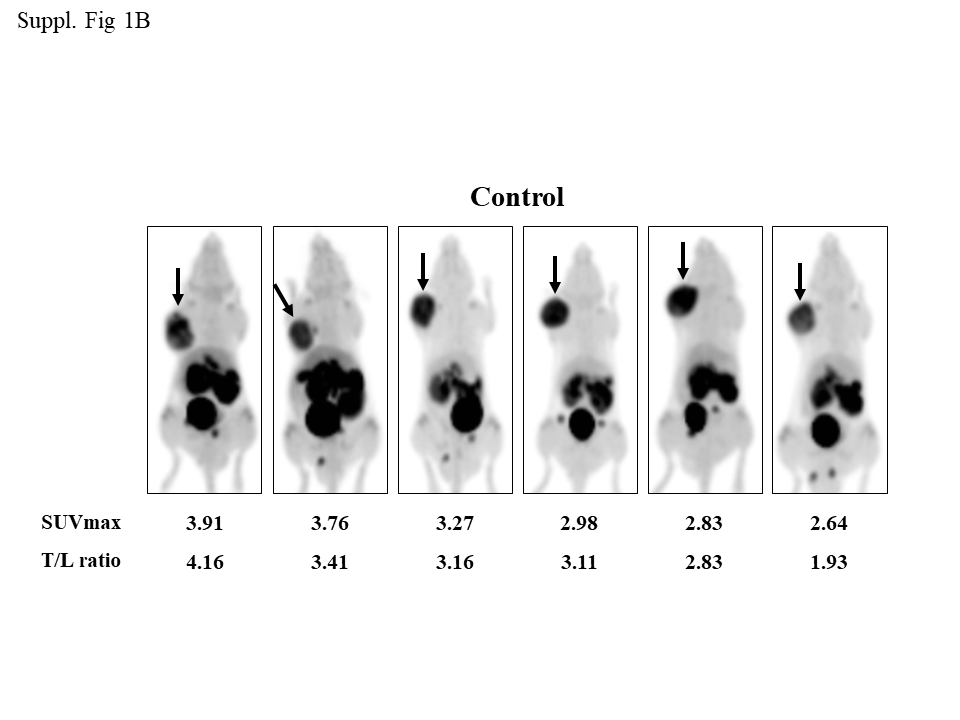

Supplement: Supplementary file 2 [file Image_2.tif]
